# The cardiac, vasomotor and myocardial branches of the baroreflex in hypotension: indications of reduced venous return to the heart

**DOI:** 10.1007/s10286-024-01076-7

**Published:** 2024-10-17

**Authors:** Gustavo A. Reyes del Paso, Casandra I. Montoro, Dmitry M. Davydov, Stefan Duschek

**Affiliations:** 1 Department of Psychology, University of Jaén, 23070 Jaén, Spain; 2 Institute of Psychology, UMIT Tirol-University of Health Sciences and Technology, Hall in Tirol, Austria

**Keywords:** Hypotension, Baroreflex, Stroke volume, Total peripheral resistance, Venous return, Heart rate variability

## Abstract

**Purpose:**

Alterations of autonomic cardiovascular control are implicated in the origin of chronic low blood pressure (BP) (hypotension), but comprehensive analysis of baroreflex function is still lacking. This study explored baroreflex function in its cardiac, vascular and myocardial branches

**Methods:**

Continuous BP was recorded at rest and during a mental arithmetic task in 40 hypotensive and 40 normotensive participants. Assessed cardiovascular variables included stroke volume (SV) (calculated by the Modelflow method), heart rate (HR), cardiac output (CO), total peripheral resistance (TPR) and heart rate variability (HRV). Baroreflex sensitivity (BRS) was calculated using the spontaneous sequence method.

**Results:**

Hypotensive participants exhibited greater BRS in the three baroreflex branches, in addition to lower SV, HR and CO and higher HRV and TPR. Reactivity for BP, HRV and CO during the stress task was reduced in hypotensive individuals. The greater cardiac BRS can explain the lower HR and higher HRV observed in hypotension, suggestive of increased vagal cardiac influences. The higher vasomotor BRS may contribute to the greater TPR observed in the hypotensive participants. Abnormal associations between myocardial BRS and SV arose, suggesting aberrant autonomic control of myocardial contractility in hypotension.

**Conclusion:**

The results indicate that hemodynamic deficits in hypotension are related to preload factors, probably triggered by hypovolemia and reduced unstressed blood reserves, resulting in lower venous return, ventricular preload and SV. In contrast, afterload mechanisms seem to work appropriately.

**Supplementary Information:**

The online version contains supplementary material available at 10.1007/s10286-024-01076-7.

## Introduction

Chronic hypotension refers to a persistent state of inappropriately low blood pressure (BP) independent of the occurrence of further pathological conditions [1]. The chronic form of hypotension is distinguished from orthostatic hypotension (namely, hemodynamic problems when assuming an upright position) and symptomatic hypotension, which occurs, for example, due to blood loss or medication use [2]. Although there is no generally recognized definition of chronic hypotension, in psychophysiological research, systolic BP (SBP) baseline values of around 100 mmHg are frequently used as an upper limit (e.g. [3–6]). Typical complaints associated with chronic hypotension include fatigue, reduced drive, dizziness, headaches and cold limbs, as well as an altered emotional state characterized by symptoms of depression, all of which significantly reduce wellbeing and quality of life [7]. Individuals with hypotension typically present with cognitive deficits, which have been ascribed to suboptimal cerebral blood flow regulation and blunted cortical activity [8–10]. Increased pain sensitivity was also reported in this population [11].

Deficient cardiovascular autonomic control is believed to play a key role in the etiology of chronic hypotension [3, 12]. Reduced cardiac sympathetic outflow may contribute to this condition. Individuals with hypotension exhibit lower stroke volume (SV) than those with normal BP, both at rest and during cognitive activity [5, 11, 13]. In addition, hypotension is associated with prolongation of the pre-ejection period (PEP) at rest, during mental activity and during sleep [5, 13–15]. SV is positively related and PEP is inversely related to β_1_-adrenergic activity; as such, these observations confirm the notion of reduced myocardial sympathetic tone in chronic hypotension. However, myocardial contractility strongly depends on the extent of blood return to the heart [16, 17]; thus, a reduction in this return and subsequent ventricular preload may also contribute to the low SV. Regarding α-adrenergic sympathetic influences, studies comparing total peripheral resistance (TPR) between hypotensive and normotensive individuals have not revealed differences [5, 11, 13]. Enhanced parasympathetic outflow may also be involved in persistently low BP. Hypotensive individuals display a lower heart rate (HR) [5, 13], suggestive of increased vagal cardiac tone [18, 19]. However, studies on the quantification of vagal cardiac influences via measurement of HR variability (HRV) revealed inconsistent results: while increased HRV during sleep supported the hypothesis of elevated parasympathetic tone [14], a lack of difference in HRV between awake individuals with low and normal BP was also reported [5, 13, 15]. The cardiac baroreflex is the main source of vagal influences to the heart [19, 20], and enhanced cardiac baroreflex sensitivity has repeatedly been described in hypotensive individuals [5, 13, 21], suggesting increased parasympathetic influences in this population.

Malfunction of the baroreflex may be a relevant factor in the genesis of chronic hypotension. This medullary reflex consists of a negative feedback loop, in which changes in activity in arterial baroreceptors resulting from BP fluctuations precipitate compensatory changes in HR, myocardial contractility and vascular tone. During an acute BP rise, the reflex inhibits β_1_-adrenergic outflow to the myocardium (reducing contractility and thus SV as its surrogate estimate) and α-adrenergic outflow to the vasculature (causing systemic vasodilatation) and increases cardiac vagal tone (reducing HR). The opposite reflex responses are elicited during BP decline [22]. According to these functions, three baroreflex subsystems can be distinguished: the cardiac branch controlling chronotropic influences (HR) through the vagus nerve, the myocardial branch controlling inotropic influences (SV) via β_1_-sympathetic fibers and the vascular branch modulating vasomotor tone (vascular resistance) through α-adrenergic sympathetic influences [23].

Functional properties of the arterial baroreflex can be noninvasively quantified through the analysis of spontaneously occurring covariation of BP with reflex-induced changes in HR, myocardial contractility and vasomotor tone. This method has most frequently been applied to describe the sensitivity of the cardiac branch of the reflex (cardiac baroreceptor reflex sensitivity [cBRS]), expressed as the change in interbeat interval (IBI) per unit of BP change [24]. Sequence analysis, which locates sequences of consecutive cardiac cycles in which the reflex is in operation, may be used for this purpose. In these periods, either a progressive SBP increase is accompanied by an increase in IBI (up sequences), or SBP decrease is accompanied by a reduction in IBI (down sequences). cBRS is defined as the slope of the regression line between changes in SBP and IBI within the detected baroreflex sequences [24, 25].

While the arterial baroreflex is regarded as the most important control system for buffering short-term BP fluctuations, research has also confirmed its involvement in the setting of tonic BP and the origin of essential hypertension [26, 27]. cBRS is inversely related to BP in the normotensive range, and a reduction of cBRS in hypertension has been repeatedly documented [26, 28]. Regarding chronic low BP, elevated cBRS has been observed at rest and under conditions of cognitive effort [5, 13, 21]. It may be that exaggerated responsiveness of the reflex leads to overcompensation of phasic BP rises, resulting in the stabilization of BP at a lower level [21].

While cBRS constitutes a well-established parameter in various fields of medical and behavioral research (e.g. [22, 29, 30]), the functions of the myocardial (m) and vascular (v) branches of the reflex have scarcely been regarded so far. Recently, a method has been proposed that enables reliable quantification of the sensitivity of these subsystems (mBRS and vBRS, respectively) [23]. This method is based on the same principle as sequence analysis of cBRS: sequences in which progressive increases in SBP are associated with decreases in SV and TPR, and vice versa (for details of the validation of the method, see [23]). While greater cBRS is associated with a lower HR and higher HRV, higher mBRS and vBRS are related to greater SV and TPR, respectively, as well as greater variability in these measures, suggesting asymmetry in the functioning of the arterial baroreflex in its myocardial and vasomotor branches: BP falls are more efficiently buffered than BP increases, with greater protection against acute BP falls than acute BP rises [23, 31].

The present study aimed to perform a comprehensive investigation of baroreflex function in chronic hypotension, addressing the cardiac, myocardial and vascular branches of the reflex. Currently available findings only demonstrate altered baroreflex-induced parasympathetic (chronotropic) influences on hypotension [5, 13, 21]. However, as reflected by the reduction of SV and enhancement of PEP, autonomic dysregulation in hypotension also involves the sympathetic nervous system [4, 5, 14]. Considering this, possible alterations of the reflex in its vasomotor and myocardial branches may contribute to the reduction in myocardial contractility and/or vasomotor tone.

Furthermore, relevant BP features related to the magnitude of the stimulation input to the baroreceptors have not yet been studied in hypotension. These include the magnitude and slope of the increase in BP during the systolic upstroke, the number of progressive SBP changes and the slope of these changes [32, 33]. Assuming a reduction of these variables, the baroreflex would receive less stimulation, which may further affect its functioning.

Based on previous reports [5, 13, 21], we expected to observe greater cBRS in hypotensive than in normotensive individuals. Given the lower SV observed in hypotension and the positive relation between mBRS and SV [23, 31], we predicted reduced mBRS in hypotensive individuals. Regarding the vasomotor branch, as previous studies did not reveal alterations in TPR in hypotension [5, 11, 13], we did not expect differences in vBRS between hypotensive and normotensive individuals. The relative impacts of preload (SV and IBI) and afterload (TPR) on the regulation of BP were evaluated by regression analysis. As results from previous studies suggested lower SV in hypotension [5, 13], we expected to observe a greater impact of SV with respect to the determination of BP in hypotensive as compared to normotensive individuals. Given the low BP values, we also expected to see a smaller stimulation input to the baroreceptors in hypotension, with a lower slope of the systolic peak and a lower number and slope of progressive SBP changes. In order to obtain a more comprehensive picture, cardiovascular recordings were carried out under resting conditions and during mental challenge. This procedure allowed evaluation of the magnitude of cardiovascular reactivity during stress conditions, where previous research pointed to blunted reactivity in chronic hypotension [3, 5, 34].

## Methods

### Participants

Forty subjects with hypotension (HT) (38 women, 2 men), defined as SBP < 100 mmHg in women and < 110 mmHg in men, participated in the study; the control group (normotension [NT]) included 40 participants (37 women, 3 men) with SBP between 115 and 140 mmHg. Due to the lack of a universally accepted definition of chronic hypotension, participants were selected based on previous psychophysiological research. The same criteria were used, for example, in previous studies on the associations of hypotension with cerebral blood flow [6, 35–37], central-nervous activity [8, 38, 39], autonomic regulation [3, 4, 6, 11, 13, 14, 21], cognitive performance [5, 34], pain sensitivity [21, 40], mood disturbance [5] and pharmacological treatment [41–44].

None of the subjects in either group suffered from a relevant physical disease or mental disorder, nor did they use any kind of medication affecting the cardiovascular or central/ peripheral nervous system. In total, 67 of the participants were university students (34 in the hypotensive sample, 33 in the control group); the remaining subjects were recruited from the workforce.

### Hemodynamic recordings

Blood pressure was recorded continuously with a Finometer Model-2 noninvasive BP monitor (Finapres Medical Systems, Amsterdam, the Netherlands). The cuff of the device was applied to the left index finger and the left hand was positioned at the level of the heart. For periodic recalibration, the device’s “Physiocal” feature [45] was in operation. Hemodynamic parameters were determined on a beat-to beat basis, based on continuous BP recordings made through the Modelflow method [46] using the Beatscope 1.1a software (Finapres Medical Systems). The Modelflow technique models the aortic blood flow waveform from the finger pulse pressure by simulating a non-linear three-element model, consisting of arterial impedance, compliance and peripheral resistance. The software computes the integral of the systolic segment of the pressure waveform to obtain beat-to-beat SV (in milliliters). Cardiac output (CO: l/min) is obtained as SV × HR. This method allows for assessment of intra-individual hemodynamic changes with high accuracy [47, 48]. Comparisons of 76 CO measurements obtained using the Modelflow method during bypass surgery in eight open-heart patients yielded a mean deviation of 2% when compared with simultaneous thermodilution measurements [46] (for further validation of the Modelflow method, see, for example, [47, 49]). TPR (dyn × s/cm5) was computed as (mean BP/CO) × 80 [18]. IBI was derived from the intervals between systolic peaks. Variability in cardiovascular measures was computed as the root mean square of successive differences (RMSSD). The RMSSD of HR provides an overall index of HRV and is closely associated with respiratory sinus arrhythmia (RSA), i.e. HRV in the frequency range of respiration [50], constituting a well-established index of parasympathetic influences on HR [18].

### Computation of baroreflex parameters

Baroreflex function was quantified using the spontaneous sequence method in the time domain [24, 51, 52]. The term “ramps” was used to designate progressive SBP changes, and the term “sequences” was used to designate reflex concomitant SBP-IBI, SBP-SV or SBP-TPR changes. Sequences of 1–3 consecutive heartbeats were localized, in which SBP increases (“up sequences”) were accompanied by increases in IBI (cardiac branch, “c”), decreases in SV (myocardial branch, “m”) or decreases in TPR (vasomotor branch, “v”); and SBP decreases (“down sequences”) were accompanied by IBI decreases and SV or TPR increases. The correlation between SBP and the corresponding output measures within a given sequence had to be > 0.85 for such changes to be considered as a reflex sequence. When a baroreflex sequence was detected, the regression line between the SBP and IBI, SV or TPR values within the sequence was computed. BRS was expressed as the change in IBI, SV or TPR per millimeters of mercury (mmHg) change in SBP, given by the slope of the regression line. BRS was calculated as the average of all detected sequences. The reflex is not always effective (i.e. not all SBP ramps are followed by compensatory changes in the output variables). The baroreflex effectiveness index (BEI), calculated as the ratio (%) between the number of SBP ramps followed by reflex changes (i.e. the number of sequences) and the total number of SBP ramps, captures this feature of the baroreflex [32, 33]. The minimum criteria for consideration of a change as progressive was 1 mmHg for SBP, 1 ms for IBI, 1 dyne s/cm^5^ for TPR (minimum change of 3 units within a sequence for these measures) and 0.4 ml for SV (minimum change of 1.2 ml). To avoid the inclusion of extreme values or artefacts, values ± 20% of the mean in each experimental period for IBI and TPR and values ± 10% of the mean for SV were rejected when computing the reflex sequences. The lower criterion values for SV result from its lower values and narrow variability range [23]. To select the time delays (measured as the lag of beats) between the SBP changes and the output measures, we tested the suitability of lags of 1–3 beats. These lags were compared in terms of the number of reflex sequences detected and the BEI values. Based on these analyses, for the cardiac branch, SBP values were paired with the immediately following IBI [52], and a 1-beat lag for the myocardial branch and 2-beat lag for the vasomotor branch were used. More detailed information on the method can be found elsewhere [23, 31]. The magnitude of the stimulation input to the baroreceptors was analyzed based on the following features: (1) slope of the increase in BP from the diastolic to the systolic points (mmHg/ms); (2) amplitude of the SBP peak (diastolic blood pressure [DBP] to SBP); (3) number of SBP ramps; and (4) slope of the SBP ramps, as indicated by the regression line between the SBP values within the ramp and time (mmHg/s) [32, 33].

### Procedure

The present study was part of a larger project dedicated to the analysis of cognitive performance, cerebral blood flow and autonomic control in hypotension [5, 13, 35, 36]. None of the data reported here were included in any previous publication. Assignment of subjects to the two study groups was carried out on the basis of BP readings taken during a screening session, which was conducted at least 1 week prior to the main experiment and again at the beginning of the experimental session. Here, after a rest period of 10 min, three sphygmomanometric BP measurements were taken in a sitting position. For this purpose, an automatically inflating BP monitor (Omron M400; Omron Healthcare, Vernon Hills, IL, USA) was used. Readings were separated by 5-min rest intervals. The mean value of the three measurements was used for group assignment. The SBP inclusion criterion had to be fulfilled in both the screening and experimental sessions.

Hemodynamic measures were recorded at rest and during mental stress induced by a serial subtraction task. During the 7-min resting phase, participants were asked to sit still, not to speak and to relax with their eyes open. The subtraction task was conducted over a 3-min period, during which subjects had to count down from 1000, subtracting 17 each time and saying the numbers out loud. They were asked to perform the task as quickly and as accurately as possible. Experimental sessions were conducted in the morning, between 8 a.m. and 11 a.m., and in the afternoon between 2 p.m. and 5 p.m. To control for circadian effects, the same number of participants from both study groups were tested in the morning and afternoon. Participants were requested not to drink alcohol or beverages containing caffeine for 3 h prior to the screening and experimental sessions. The study was approved by the Board for Ethical Questions in Science of the University of Innsbruck, Austria, and all participants provided written informed consent.

### Data analysis

Mean values across the resting and mental stress conditions were computed for each variable. Analysis of variance (ANOVA) models were constructed with experimental group (hypotensive vs. control group) as the between-subjects factor and Condition (rest vs. mental stress) as the within-subjects factor. Post-hoc *F*-tests were used to analyze Condition separately in each group when a significant Group × Condition interaction effect was present. Comparisons between the “up” and “down” sequences were computed using a t-test. Associations between cardiovascular variables within each study group were analyzed by Pearson correlation and multiple linear regression analyses (“enter” method). As all analyses were conducted to test specific hypotheses, no adjustments for repeated statistical testing were made, as recommended [53]. Previous studies comparing parameters of autonomic control between HT and NT individuals revealed effect sizes (Cohen’s d) of between 0.3 and 0.5. Based on an* α* level of 0.05, an estimated effect size of 0.40 and a beta error of 20%, a sample size of 34 participants per group appeared optimal to achieve a statistical power of 80%.

## Results

Data for BP and HR, oscillometrically assessed immediately before the experimental session, are presented in Table 1, together with the age and BMI of the participants. The HT participants not only had lower BP and HR than the NT participants, but also had lower BMI. Results on the amplitude and slope of the systolic peak, the number and slope of SBP ramps, BEI, differences between the “up” and “down” sequences and the correlations of the input stimulation to the baroreceptors with BRS and BEI are given in the Electronic Supplementary Material (ESM).

**Table 1:** Age, body mass index, systolic blood pressure, diastolic blood pressure and heart rate values of participants as evaluated during the screening period on the day of the experimental session

Patient characteristics	Mean ± SD	*F*	*p*
*Age (years)*			
HT	22.95 ± 3.07	0.98	.38
NT	22.97 ± 4.02		
*BMI (kg/m*^*2*^)			
HT	20.52 ± 2.14	3.71	.03
NT	21.92 ± 2.51		
*SBP (mmHg)*			
HT	94.48 ± 5.59	209.91	< .01
NT	122.29 ± 6.41		
*DBP (mmHg)*			
HT	62.23 ± 4.71	99.15	< .01
NT	79.12 ± 5.84		
*HR (bpm)*			
HT	67.90 ± 11.33	10.30	< .01
NT	79.91 ± 12.27		

*BMI* Body mass index, * DBP* diastolic blood pressure, * HR* heart rate, * HT* hypotensive (group), * NT *normotensive (control group), * SBP* systolic blood pressure, * SD* standard deviation

### Group differences in cardiovascular variables and effects of the arithmetic task

Table 2 presents the means and standard deviations (SD) of the cardiovascular variables. In comparison to the NT group, the HT group showed lower SBP (*F*_(1, 78)_ = 54.67, *p* < 0.001, ${\eta }_{p}^{2}$ = 0.41) and DBP (*F(*_1, 78)_ = 25.09, *p* < 0.001, ${\eta }_{p}^{2}$ = 0.24); lower HR (*F*_(1, 78)_ = 28.90, *p* < 0.001, ${\eta }_{p}^{2}$ = 0.27) and greater HRV (*F*_(1, 78)_ = 13.75, *p* < 0.001, ${\eta }_{p}^{2}$ = 0.15); higher TPR (*F*_(1, 78)_ = 5.54, *p* = 0.021, ${\eta }_{p}^{2}$ = 0.07) and TPR variability (*F*_(1, 78)_ = 13.20, *p* < 0.001, ${\eta }_{p}^{2}$ = 0.15); lower SV (*F*_(1, 78)_ = 16.56, *p* < 0.001, ${\eta }_{p}^{2}$ = 0.18); and lower CO (*F*_(1, 78)_ = 54.91, *p* < 0.001, ${\eta }_{p}^{2}$ = 0.41. Statistical control of HR resulted in a larger group difference in SV (*F *= 21.57, ${\eta }_{p}^{2}$ = 0.22). While there was no main effect of Group on the product of SV × TPR (*F*_(1, 78)_ = 1.26, *p* = 0.27, ${\eta }_{p}^{2}$ = 0.02), the product of CO × TPR was lower in the HT group than in the NT group (*F*_(1, 78)_ = 39.33, *p* < 0.001, ${\eta }_{p}^{2}$ = 0.34).

**Table 2:** Cardiovascular variables during the baseline and mental arithmetic task periods

Cardiovascular variables	Baseline	Arithmetic task
*SBP (mmHg)*		
HT	96.21 ± 14.12	100.32 ± 13.58
NT	113.75 ± 12.74	124.40 ± 13.21
*SBPv (mmHg)*		
HT	3.15 ± 1.36	5.10 ± 1.40
NT	3.23 ± .83	5.49 ± 2.04
*DBP (mmHg)*		
HT	41.50 ± 10.48	47.37 ± 9.28
NT	51.66 ± 11.67	60.41 ± 11.45
*HR (bpm)*		
HT	64.01 ± 8.68	70.03 ± 8.72
NT	74.57 ± 11.68	83.10 ± 11.47
*HRV (ms)*		
HT	58.79 ± 22.77	58.15 ± 21.73
NT	45.10 ± 22.30	39.64 ± 15.75
*SV (ml)*		
HT	73.98 ± 18.98	72.73 ± 16.29
NT	89.01 ± 19.77	89.53 ± 16.79
*SVv (ml)*		
HT	4.73 ± 1.26	4.35 ± 1.22
NT	4.79 ± 1.30	4.84 ± 1.15
*CO (l)*		
HT	4.67 ± 1.08	5.05 ± 1.13
NT	6.59 ± 1.56	7.40 ± 1.59
*TPR (dyne s/cm5)*		
HT	798.08 ± 237.85	797.23 ± 190.75
NT	691.73 ± 222.65	692.18 ± 172.56
*TPRv (dyne_s/cm5)*		
HT	51.14 ± 21.10	59.48 ± 21.31
NT	36.76 ± 20.76	43.40 ± 16.21

Values in table are presented as the mean ± standard deviation

*CO* Cardiac output, * DBP* diastolic blood pressure, * HR* heart rate, * HRV* HR variability HT hypotensive group, * NT* normotensive (control) group, * SBP* systolic blood pressure, * SBPv* SBP variability, * SV* stroke volume, * SVv* SV variability, * TPR* total peripheral resistance, * TPRv* TPR variability

SBP and DBP increased during the arithmetic task in both groups (*F*_(1, 78)_  = 49.91, *p* < 0.001, ${\eta }_{p}^{2}$ = 0.39 for SBP; *F*_(1, 78)_  = 125.25, *p* < 0.001, ${\eta }_{p}^{2}$ = 0.62 for DBP); furthermore, a Condition × Group interaction arose (*F*_(1, 78)_  = 9.81, *p* = 0.002, ${\eta }_{p}^{2}$ = 0.11 for SBP; *F*_(1, 78)_ = 4.82, *p* = 0.031, ${\eta }_{p}^{2}$ = 0.06 for DBP). Although significant in both groups, the increase in SBP and DBP was lower in the HT group (*F*_(1, 39)_ = 11.24, *p* = 0.002, ${\eta }_{p}^{2}$ = 0.22 for SBP; *F*_(1, 39)_ = 32.39, *p* < 0.001, ${\eta }_{p}^{2}$ = 0.45 for DBP) than in the NT group (*F*_(1, 39)_ = 39.61, *p* < 0.001, ${\eta }_{p}^{2}$ = 0.50 for SBP; *F*_(1, 39)_ = 119.34, *p* < 0.001, ${\eta }_{p}^{2}$ = 0.75 for DBP).

SBP variability increased during the task (*F*_(1, 78)_ = 99.62, *p* < 0.001, ${\eta }_{p}^{2}$ = 0.56), regardless of group. This also applied to HR (*F*_(1, 78)_ = 127.33, *p* < 0.001, ${\eta }_{p}^{2}$ = 0.62). Although the increase was somewhat larger in the NT group (8.53 bpm) than in the HT (6.03 bpm) group, the Group × Condition interaction did not reach significance (*F*_(1, 78)_ = 3.77, *p* = 0.06, ${\eta }_{p}^{2}$ = 0.06). While HRV did not change during the task (*F*_(1, 78)_ = 3.27, *p* = 0.07, ${\eta }_{p}^{2}$ = 0.04), a Group × Condition interaction was seen (*F*_(1, 78)_ = 4.27, *p* = 0.041, ${\eta }_{p}^{2}$ = 0.46). HRV significantly decreased in the NT group (*F*_(1, 39)_ = 4.39, *p* = 0.043, ${\eta }_{p}^{2}$ = 0.10) but not in the HT group (*F*_(1, 39)_ = 0.09, *p* = 0.77, ${\eta }_{p}^{2}$ = 0.002).

For SV and TPR, no main or interaction effects were observed. TPR variability increased during the task (*F*_(1, 78)_ = 23.79, *p* < 0.001, ${\eta }_{p}^{2}$ = 0.23) irrespective of group. However, a Group × Condition interaction effect arose for SV variability (*F*_(1, 78)_ = 4.07, *p* = 0.047, ${\eta }_{p}^{2}$ = 0.05). SV variability decreased during the task in the HT group (*F*_(1, 78)_ = 6.59, *p* = 0.014, ${\eta }_{p}^{2}$ = 0.14) but not in the NT group (*F*_(1, 78)_ = 0.09, *p* = 0.77, ${\eta }_{p}^{2}$ = 0.002). Finally, CO increased during the task (*F*_(1, 78)_ = 37.22, *p* < 0.001, ${\eta }_{p}^{2}$ = 0.32), with an additional Group × Condition interaction (*F*_(1, 78)_ = 4.90, *p* = 0.030, ${\eta }_{p}^{2}$ = 0.06). Although significant in both groups, the CO increase was smaller in the HT group (*F*_(1, 39)_ = 18.24, *p* < 0.001, ${\eta }_{p}^{2}$ = 0.32) than in the NT group (*F*_(1, 39)_ = 21.79, *p* < 0.001, ${\eta }_{p}^{2}$ = 0.36).

### Prediction of blood pressure and stroke volume

Table 3 shows the results of the regression analysis of the prediction of SBP from IBI, SV and TPR in both groups. While all three variables were significant predictors in this analysis, separate correlations suggested that SBP was more closely associated with SV than with IBI and TPR in the HT group.

**Table 3:** Results of the regression analysis (enter method) for the prediction of systolic blood pressure from interbeat interval, stroke volume and total peripheral resistance in the hypotensive and normotensive groups during the baseline and arithmetic task periods

Cardiovascular variables	Baseline^a^				Arithmetic task^a^			
	*β*	*t*	*p*	*r*	*Β*	*t*	*p*	*r*
*HT group*								
IBI	− 0.39	− 3.65	< 0.01	0.13	− 0.56	− 6.12	< 0.01	0.01
SV	1.16	9.25	< 0.01	0.74	1.25	11.97	< 0.01	0.66
TPR	0.51	4.31	< 0.01	− 0.18	0.81	7.74	< 0.01	−0 .02
*NT group*								
IBI	− 0.85	− 6.40	< 0.01	− 0.27	− 0.78	− 8.70	< 0.01	− 0.49
SV	1.12	6.77	< 0.01	0.23	1.06	7.65	< 0.01	0.04
TPR	1.16	5.51	< 0.01	0.11	1.15	8.50	< 0.01	0.28

*HT* Hypotensive, * IBI* interbeat interval, * NT* normotensive (control), * SV* stroke volume, * TPR* total peripheral resistance

^a^*Β *, Standardized beta coefficient from the overall model;* r*, Person correlation for each individual variable

HRV positively predicted SV variability in both groups. However, the association was stronger in the HT group (*β* = 0.59, *t* = 4.55, *p* < 0.001 at baseline (resting); *β* = 0.65, *t* = 5.23, *p* < 0.001 during the task) than in the NT group (*β* = 0.28, *t* = 1.82, *p* = 0.08 during baseline; *β* = 0.45, *t* = 3.11, *p* = 0.004 during the task). HRV positively predicted SV in the HT group (*β* = 0.61, *t* = 4.73, *p* < 0.001 at baseline; *β* = 0.42, *t* = 2.87, *p* = 0.01 during the task) but not in the NT group (*β* = 0.004 at baseline; *β* = 0.14 during the task, ps > 0.3). cBRS also positively predicted SV in the HT group (*β* = 0.58, *t* = 4.42, *p* < 0. 001 at baseline; *β* = 0.45,=3.08, *p* = 0.004 during the task) but not in the NT group (*β* = − 0.01, *t* = − 0.03, *p* = 0.98 at baseline; *β* = 0.04, *t* = 0.27, *p* = 0.79 during the task). Finally, IBI positively predicted SV in the HT group at baseline (*β* = 0.38, *t* = 2.76, *p* = 0.02; *β* = 0.28, *t* = 1.81, *p* = 0.08 during the task) but not in the NT group (*β* = 0.14, *t* = 0.84, *p* = 0.41 at baseline; *β* = 0.17, *t* = 1.07, *p* = 0.29 during the task).

### Baroreceptor reflex sensitivity

**Fig. 1:**
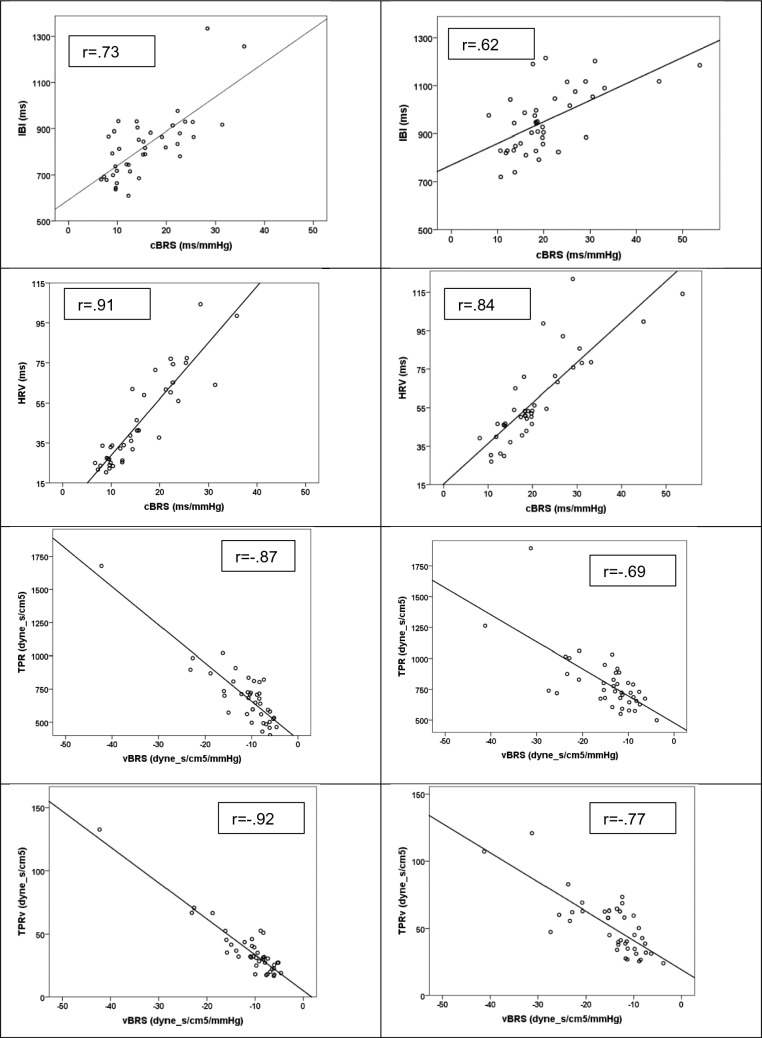
Scatterplot and regression lines in the normotensive (left) and hypotensive (right) groups displaying the relationship between cardiac baroreflex sensitivity (*cBRS*) and vasomotor baroreflex sensitivity (*vBRS*) and their output variables (inter-beat interval [*IBI*] and heart rate variability [*HRV*] for the cardiac branch, and total peripheral resistance [*TPR*] and TPR variability [*TPRv*] for the vasomotor branch) during the baseline period

cBRS was greater in the HT group than in the NT group (*F*_(1, 78)_ = 12.45, *p* = 0.001, ${\eta }_{p}^{2}$ = 0.14) and decreased during the task (*F*_(1, 78)_ = 94.98, *p* < 0.001, ${\eta }_{p}^{2}$ = 0.55), regardless of group (Table 4). Associations of cBRS with IBI and HRV were similar in both groups at baseline (Fig. 1) and during the task period (*r* = 0.65 and *r* = 0.84 in the HT group; *r* = 0.67 and *r* = 0.78 in the NT group, for IBI and HRV during the task, respectively, all* p* < 0.001).

**Table 4:** Baroreceptor reflex sensitivity during the baseline and the mental arithmetic task periods for the cardiac, vasomotor and myocardial branches of the baroreflex in the hypotensive and normotensive groups

Cardiovascular variables	Baseline	Arithmetic task
*cBRS (ms/mmHg)*		
HT	20.62 ± 9.02	14.03 ± 4.86
NT	15.77 ± 7.17	9.65 ± 3.77
*vBRS (dyne s/cm5/mmHg)*		
HT	−14.67 ± 7.47	−11.78 ± 4.67
NT	−11.16 ± 6.72	−9.49 ± 4.12
*mBRS (ml/mmHg)*		
HT	−2.68 ± 1.33	−2.76 ± 1.55
NT	−2.38 ± 0.91	−1.98 ± 0.73

Values in table are presented as the mean ± standard deviation

*BRS* Baroreceptor reflex sensitivity, * c, v, m* cardiac, vasomotor, and myocardial branches of baroreflex, respectively, * HT* hypotensive (group), * NT* normotensive (control group)

vBRS was greater in the HT group than in the NT group (*F*_(1, 78)_  = 6.54, *p* = 0.01, ${\eta }_{p}^{2}$ = 0.08), and it decreased during the task (*F*_(1, 78)_ = 11.20, *p* = 0.001, ${\eta }_{p}^{2}$ = 0.13) regardless of group. vBRS was negatively associated with TPR and TPR variability during both baseline (Fig. 1) and the task (*r* = − 0.70 and *r* = −0.72 in the HT group; *r* = −0.73 and *r* = − 0.64 in the NT group, for TPR and TPR variability during the task, respectively; all* p* < 0.001). Given the negative values of vBRS, greater sensitivity was related to higher TPR and TPR variability.

**Fig. 2:**
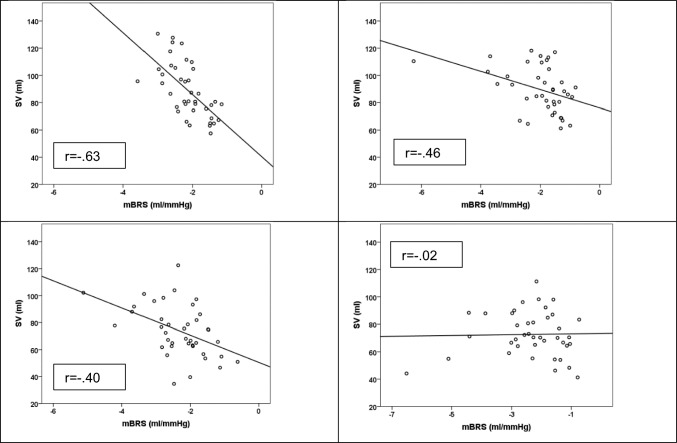
Scatterplot and regression lines in the normotensive (left) and hypotensive (right) groups displaying the relationship between myocardial baroreflex sensitivity (*mBRS*) and stroke volume (*SV*) during the baseline period (top) and the arithmetic task period (bottom)

Greater mBRS was seen in the HT than in the NT group (*F*_(1, 78)_ = 7.99, *p* = 0.01, ${\eta }_{p}^{2}$ = 0.09). No main effect of Condition (*F*_(1, 78)_ = 0.79, *p* = 0.38, ${\eta }_{p}^{2}$ = 0.010) or interaction (*F*_(1, 78)_ = 1.70, *p* = 0.20, ${\eta }_{p}^{2}$ = 0.02) effect arose. HRV negatively predicted mBRS, with greater weight in the HT group (*β* = − 0.55, = − 4.00, *p* < 0.001 during baseline; *β* = − 0.33, *t* = − 2.17, *p* = 0.04 during the task) than in the NT group (*β* = − 0.32, *t* = − 2.07, *p* = 0.05 during baseline; *β* = − 0.10, *t* = − 0.59, *p* = 0.56 during the task). mBRS was negatively associated with SV (Fig. 2) and SV variability during baseline (*r* = − 0.56 in the HT group; *r* = − 0.71 in the NT group for SV variability; all* p* < 0.001). However, during the task, correlations were only significant in the NT group (*r* = − 0.12, not significant, in the HT group; *r* = −0.38,* p* < 0.05, in the NT group for SV variability) (see Fig. 2 for the correlations with SV). Given the negative values of mBRS, greater mBRS was related to higher SV and SV variability.

## Discussion

As expected, this study revealed lower SV, HR and CO in individuals with chronic hypotension than in normotensive controls. In addition, hypotensive individuals exhibited higher TPR and HRV, as well as greater BRS in all three branches of the baroreflex. According to these observations, reduced myocardial contractility, together with increased cardiac vagal activity and exaggerated arterial baroreflex function, may be the principal components of the autonomic phenotype associated with chronic hypotension.

This study confirmed previous findings of lower SV and HR in hypotension, leading to reduced CO [5, 11, 13]. Importantly, SV was far smaller in the HT group despite the lower HR. Bradycardia commonly increases SV due to a longer diastolic filling time, which raises end-diastolic volume and stretches cardiac muscle fibers, thereby increasing contractility and SV (i.e. the Frank–Starling mechanism; see, e.g., [16, 17]). This finding corroborates inotropic alterations in chronic hypotension, with a shift in the relationship between the force of cardiac contraction and fiber length for the same blood volume, finally leading to reduced SV. The CO reduction may result in diminished cerebral blood flow and organ blood perfusion, which in turn might account for some of the complaints that typically accompany chronically low BP [10–12, 14].

Unlike previous studies, we also found higher HRV and TPR in hypotensive than normotensive individuals (see [4, 5, 11, 13, 21] for inconsistent results pertaining to HRV and TPR in hypotension). The higher HRV (i.e. RMSSD) in the hypotensive sample reflects an increased vagal cardiac influence [18], which contributes to the HR reduction. This result is in line with the results reported by Covassin et al. [14], which revealed increased RMSSD during sleep in hypotension. High HRV in hypotension is physiologically coherent with high cBRS, as cBRS is the main source of vagal cardiac efferent influences on HR and a core factor determining HRV [19, 20, 54]. The higher TPR observed in our HT group points toward greater activation of the renin–angiotensin system and α-adrenergic influences, compensating for the CO reduction. This finding contrasts with previous reports of a lack of differences in TPR between hypotensive and normotensive samples [5, 11, 13, 21]. However, it is partially coherent with the observation of Covassin et al. [14] pertaining to nocturnal autonomic control in hypotension; these authors observed a progressive TPR rise during the second half of the night, which was interpreted as a compensatory response to the progressive drop in CO. The comparison of the product of SV × TPR between the groups accords with the view that the lower SV was compensated for by the TPR increase. However, as suggested by the reduced product of CO × TPR, the TPR elevation in the HT group was not sufficient to fully compensate for the lower CO. Taken together, these results suggest that cardiac preload factors (i.e. lower SV and HR) are core features of the cardiovascular dysregulation characterizing hypotension.

The higher HR and CO seen during the arithmetic task with respect to baseline reflects stress-induced sympathetic activation and parasympathetic withdrawal [11]. Importantly, a decrease in HRV during the task was only seen in the NT group. While vagal activity decreased during stress in normotensive individuals, thereby improving the ability to cope with task demands, in hypotensive individuals vagal activity was maintained at a high level, thus preventing optimal energy mobilization and efficient coping (see [4, 13] for equivalent results).

SV and TPR remained largely unchanged during the arithmetic task. Previous findings concerning these variables during stress are inconclusive, suggesting that the type and intensity of mental stress moderate the effects [5, 11, 13, 21]. While TPR variability increased during the task, irrespective of group, SV variability decreased only in the HT group. Moreover, the extent of the task-induced increase in BP was smaller in hypotensive than normotensive individuals. This is consistent with previous reports of blunted BP reactivity in chronic hypotension, which has been interpreted in terms of insufficient hemodynamic adjustment to situational requirements [3, 5, 13, 21, 34]. Reduced BP reactivity may also be involved in the deficient cerebral blood flow adjustment that was repeatedly observed in hypotension [6, 10, 55]. Regardless of group, SBP variability also increased during the task, leading to stronger input stimulation to the baroreceptors (i.e. increases in the number and slope of SBP ramps).

The results of the regression and correlation analyses for the prediction of SBP suggest a disproportionally higher importance of SV in determining SBP in the HT group at rest and during the task, in addition to a relatively small influence of IBI and TPR. The positive association between HRV and SV in the HT group suggests that HRV at the frequency range of respiration (i.e. RSA) plays a relevant role in hemodynamic homeostasis in hypotension. This influence may be related to the modulation of diastolic ventricular filling time by RSA and changes in the blood flow pressure gradient generated during respiration (i.e. the breathing “pump”), as modulators of venous blood return (see below).

Regarding the stimulation input to the baroreceptors, all assessed features in our study showed lower levels in hypotension: the slope and amplitude of the SBP peak, number of SBP ramps and slope of the SBP ramps (Supplementary Material S1). This underlines the occurrence of a lower level of baroreceptor stimulation in hypotension. Differences in excitatory input from baroreceptor afferents may lead to reorganization of neural processing within the nucleus of the solitary tract and related brain stem nuclei, thereby compensating for chronically reduced baroreceptor load. Although the slope of the SBP ramps was positively associated with the BEI, both the number of SBP ramps and their slope were inversely associated with BRS (see Supplemental Material S4), suggesting modifications in the baroreflex gain as a function of the level of baroreceptor stimulation.

Before discussing the BRS results, it should be noted that the global effect of the baroreflex on the output measures aims to stabilize BP via the compensatory activity changes in the three branches [23]. Sequences of BP rises activate the cardiac branch, which elicits an increase in vagal cardiac activity, whereas sequences of BP falls activate both the vasomotor (eliciting increases in α-adrenergic influences) and myocardial (eliciting increases in β_1_-adrenergic influences) branches. In contrast, the cardiac branch is inhibited during BP falls, and the vasomotor and myocardial branches are inhibited during BP rises. Thus, overall, a negative relation was established between cBRS and HR, and a positive one between vBRS and TPR and between mBRS and SV.

The numbers of reflex sequences detected in the three baroreflex branches were sufficient to compute estimates of BRS (see Supplementary Material S2). As previously observed, the number of sequences was greater in the cardiac branch than in the vasomotor and myocardial branches [23]. Overall, fewer reflex sequences arose in the HT group than in the NT group, and this difference was especially large in the myocardial branch. Apparently, low tonic BP was associated with fewer BP oscillations and reflex sequences. Furthermore, greater overall BRS, and thus improved compensation of BP oscillations, may lead to more stable BP in hypotension. During the task period, the NT group exhibited more “up” than “down” SBP ramps (congruent with the task-related BP increase); in contrast, the HT group showed more “down” than “up” SBP ramps (see Supplementary Material S2), reflecting deficient hemodynamic adjustment during the task.

Replicating previous findings [5, 11, 13, 21], cBRS was markedly higher in our hypotensive sample. The greater cBRS can explain the lower HR and higher HRV observed in the HT group, as cBRS is the main generator of parasympathetic cardiac influences [19, 20, 23]. cBRS was lower during the task than during the rest period; this is a common observation under conditions of mental activity and other laboratory stressors [20, 28, 54]. cBRS attrition implies a reduced vagal buffering effect of the baroreflex, which facilitates stress-induced HR, CO and BP increases, enhancing the oxygen supply to body tissues during increased metabolic demands. In turn, this may enhance the adaptation of motor and neural processes during acute stress [22]. A reduction in cBEI was seen in the NT but not in the HT group (see Supplementary Material S3.1, S5.1). cBEI reduction during stress supports the decrease of vagal influences (i.e. HR rise and HRV reduction), allowing BP to increase and improving the ability to cope with acute demands in the normotensive situation.

vBRS was greater in the HT group than in the NT group. Given that vBRS is a major determinant of TPR level and variability (with a positive relationship), the greater vBRS in the HP group can explain the higher TPR and TPR variability observed in that group. vBRS decreased during the task, irrespective of group; in this case, lower reflex sensitivity causes a stress-related reduction of TPR and TPR variability. This may constitute a homeostatic mechanism preventing harmful BP increases due to the activation of top-down influences on cardiovascular functioning during stress. vBEI in the “down” sequences increased in the HT group during the task (see Supplementary Material S3.2 and S5.2), suggesting that the reflex is more effective in buffering BP decreases via compensatory increases in vasomotor tone in hypotension during stress. Both the greater vBRS and the increase in vBEI in the “down” sequences during the task suggest that the vasomotor branch of the baroreflex works properly in hypotensive individuals, serving as a compensatory TPR-elevating mechanism preventing a further BP decrease.

This study also revealed greater mBRS in the HT group than in the NT group, especially in the “down” sequences (Supplementary Material S5.3), which implies that the baroreflex buffered acute BP falls by increasing myocardial contractility with greater sensitivity in hypotensive individuals. No effect of the task on mBRS was observed; this is in line with previous observations of a lack of effect of psychological challenges on mBRS [23, 31]. However, the result contrasts with the observation of lower mBEI in the HT than in the NT group (Supplementary Material S3.3). Although the reflex compensated BP fluctuations with greater changes in SV in the HT group, especially during BP falls, it was less effective overall in eliciting SV responses to these BP changes. Previous studies in healthy individuals demonstrated positive associations between mBRS and SV, both at baseline and during stress [23]. However, this association was not seen in our HT group during the task period; moreover, during baseline, the correlation was smaller in the HT than in the NT group.

Taken together, the group comparison points toward hypotension-related alterations in baroreflex control of myocardial contractility, including: (1) a lower number of baroreflex sequences; (2) smaller mBEI; (3) decoupling between the slope of SBP ramps and mBEI (Supplementary Material S4); and (4) a weaker correlation between mBRS and SV. These observations suggest the presence of another, non-baroreflex, mechanism that affects SV regulation in hypotension, reducing the influence of baroreflex on the control of myocardial contractility.

Furthermore, the findings indicate that the main hemodynamic alteration in hypotension is related to preload mechanisms, with downregulation of cardiac inotropy, whereas afterload factors seem to work appropriately. Several hypotheses may be considered to explain the decreased SV and myocardial contractility. The first, and most obvious, interpretation involves a decrease in sympathetic β_1_-adrenegic influences on the myocardium in hypotension. This has been previously suggested by the finding of a larger PEP in hypotension, which is a well-established contractility marker [5, 13]. However, it should be considered that myocardial contractility strongly depends on blood return and ventricular preload (i.e. the Frank-Starling mechanism [16, 17]).

A relevant factor that determines venous return, and thus myocardial preload and SV, is the capacitance of the venous system and the volume of unstressed blood located in reservoirs and not recruited into circulation. A deficit in unstressed blood in vein reservoirs may reduce blood return to the right atrium and ventricular filling (end-diastolic volume), which in turn reduces myocardial contraction strength [16, 17]. Decreased hydration and deficits in overall blood volume are related to low venous return and SV [56–59]. Chronic hypotension may be associated with low-grade hypohydration/hypovolemia and smaller unstressed peripheral reservoirs and central volume reserves. The positive association between HRV and SV in our HT group points toward this possibility. This correlation may reflect conjunct effects of RSA and the so-called breathing pump [60] on blood return to the heart. Effects of RSA on ventricular filling time occur synchronously with changes in central pressure gradients (between the peripheral circulation and the right atrium) during respiration, which promotes a smoothing of blood flow pulsations. In this mechanism, periods of extended cardiac filling are coupled with periods of decreased blood volume return during expiration, and shortened periods of cardiac filling are coupled with periods of increased blood volume return during inspiration.

Higher vagal tone (i.e. HRV) and a longer heartbeat duration (i.e. IBI) imply a longer ventricular filling time and, therefore, higher ventricular preload and SV. Lower availability of unstressed blood reservoirs, especially in the lungs, muscles, skin and splanchnic beds, may increase the effect of RSA and the respiratory pump on blood return. Smaller blood volume reserves decelerate filling of the heart chambers, strengthening the association between HRV and SV. In accordance with this notion, a recent study revealed correlations between HRV and SV during standing (a body position associated with low availability of central blood volume) but not during lying down (a posture related to high availability of central blood volume) (unpublished results). In hypotension, HR fluctuations in the respiratory range might be transferred to oscillations in venous return under conditions of low blood volume; hemodynamic mechanisms cannot sufficiently adjust stressed blood volume by recruiting unstressed reserves. The availability of unstressed central blood volume reserves in normotensive individuals promotes a smoothing of blood flow fluctuations to the heart, which is reflected by the lack of a correlation between HRV and SV.

The putative role of reduced venous blood return in hypotension points to the necessity of studying the function of cardiopulmonary (low pressure) baroreceptors in this condition. The implications of the cardiopulmonary baroreflex in hypotension should be considered together with that of arterial baroreflex mechanisms. Cardiopulmonary baroreceptors may adjust (“reset”) to low central hydrostatic pressure (i.e. low central venous and atrio-ventricular filling pressures), leading to reduced sympathetic outflow and decreased renal fluid retention [59].

Some methodological limitations of this study warrant discussion. The criteria applied during recruitment of hypotensive individuals, i.e., SBP < 100 mmHg in women and < 110 mmHg in men, may be relatively lenient [3, 14]. Recently proposed definitions include lower cut-off values and also take into account the DBP value. For example, the US National Heart, Lung and Blood Institute (NHLBI) suggested 90 and 60 mmHg as SBP and DBP limits, respectively [61]. The application of more strict criteria would potentially reveal a different pattern of autonomic dysregulation. Another limitation concerns the method used to derive SV from BP waveforms, i.e. the Modelflow method [46]. While this method allows relatively precise assessment of intra-individual hemodynamic changes (as the progressive changes used to analyze the properties of the baroreflex), its precision in the assessment of inter-individual differences in hemodynamic function is lower [47]. However, comparisons between simultaneous CO measurements using Modelflow and thermodilution techniques during different physiological conditions during open-heart surgery only yielded a mean error ranging from −0.1 to 0.4 l/min, with an average error of 0.1 l/min (2%) [46].

In conclusion, this study demonstrated greater sensitivity of the baroreflex in the cardiac, vasomotor and myocardial branches in individuals with chronic hypotension. Greater cBRS can partially explain the lower HR and higher HRV observed in hypotension, suggestive of increased vagal cardiac influences. Moreover, higher vBRS may explain the greater TPR and TPR variability observed in this condition. The lower number of reflex sequences in the myocardial branch, the smaller mBEI and the low or completely absent correlation between mBRS and SV suggest aberrant autonomic control of myocardial contractility in chronic hypotension. The hemodynamic alterations in hypotension are related to preload factors, probably triggered by hypovolemia and reduced unstressed blood reserves, resulting in reduced venous return and myocardial contractility, whereas afterload mechanisms work appropriately.

## Supplementary Information

Below is the link to the electronic supplementary material.

Supplementary file1 (DOCX 62 KB)

## Data Availability

The data of this study are available from the corresponding author upon request.
