# Correction: The cardiac, vasomotor and myocardial branches of the baroreflex in hypotension: indications of reduced venous return to the heart

**DOI:** 10.1007/s10286-025-01170-4

**Published:** 2025-10-27

**Authors:** Gustavo A. Reyes del Paso, Casandra I. Montoro, Dmitry M. Davydov, Stefan Duschek

**Affiliations:** 1 Department of Psychology, University of Jaén, 23070 Jaén, Spain; 2 Institute of Psychology, UMIT Tirol-University of Health Sciences and Technology, Hall in Tirol, Austria

**Correction: Clinical Autonomic Research** 10.1007/s10286-024-01076-7

In this article, the 3rd author name was published as Dmitry M. Daydov instead of Dmitry M. Davydov.

The original article has been corrected.

