# Supplementary material for: The cardiac, vasomotor and myocardial branches of the baroreflex in hypotension: indications of reduced venous return to the heart
Source: Clin Auton Res. 2024 Oct 17;35(1):87–99. doi: 10.1007/s10286-024-01076-7 (PMC11937168; doi:10.1007/s10286-024-01076-7)
Supplement: Supplementary file 1 — Supplementary file1 (DOCX 62 KB) [file 10286_2024_1076_MOESM1_ESM.docx]

**Supplemental Material**

**S1. Stimulation input to baroreceptors**

**S1.1. Slope and amplitude of the systolic peak**

The slope of the increase in BP during the systolic upstroke was greater in the NT than in the HT group (F(1, 78) = 27.52, p < .001, $\eta_{p}^{2}$ = .26). This measure increased between baseline and task periods (F(1, 78) = 25.16, p < .001, $\eta_{p}^{2}$ = .24) but with a difference between groups (interaction: F(1, 78) = 6.71, p = .01, $\eta_{p}^{2}$ = .08). The increase was significant in the NT group (F(1, 78) = 24.04, p < .001, $\eta_{p}^{2}$ = .38) and not in the HT group (F(1, 39) = 3.70, p =.06, $\eta_{p}^{2}$ = .09). The amplitude of the BP peak was larger in the NT group than in the HT group (F(1, 78) = 22.00, p < .001, $\eta_{p}^{2}$ = .22) and increased between baseline and task periods (F(1, 78) = 15.46, p < .001, $\eta_{p}^{2}$ = .17) irrespective of group (interaction: F(1, 78) = 2.64, p = .11, $\eta_{p}^{2}$ = .03) (Table S1).

**S1.2. Number and slope of systolic blood pressure (SBP) ramps**

The total number of SBP “up” ramps per minute (followed or not followed by reflex modulation) was higher in the NT than in the HT group (F(1, 78) = 41.59, p < .001, $\eta_{p}^{2}$ = .35) and increased between baseline and task periods (F(1, 78) = 52.33, p < .001, $\eta_{p}^{2}$ = .40) irrespective of group (interaction: F(1, 78) = 2.72, p = .10, $\eta_{p}^{2}$ = .03). The total number of SBP “down” ramps per minute was also greater in the NT than in the HT group (F(1, 78) = 11.14, p = .001, $\eta_{p}^{2}$ = .13) but did not change between baseline and task (F(1, 78) = 0.04, p =.85; interaction: F(1, 78) = 2.70, p = .10) (Table S1).

The slope of the reflex SBP “up” ramps was greater in the NT than in the HT group (F(1, 78) = 9.66, p = .003, $\eta_{p}^{2}$ = .11). The slope increased between baseline and task periods (F(1, 78) = 145.50, p < .001, $\eta_{p}^{2}$ = .65) with a difference between groups (interaction: F(1, 78) = 4.93, p = .03, $\eta_{p}^{2}$ = .06). Though significant in both groups, the increase was stronger in the NT (F(1, 39) = 101.18, p < .001, $\eta_{p}^{2}$ = .72) than in the HT group (F(1, 39) = 48.83, p < .001, $\eta_{p}^{2}$ = .56). The slope of the reflex SBP “down” ramps was also greater in the NT than in the HT group (F(1, 78) = 22.50, p < .001, $\eta_{p}^{2}$ = .22). It increased between baseline and task periods (F(1, 78) = 150.42, p <.001, $\eta_{p}^{2}$ = .66) with a difference between groups (interaction: F(1, 78) = 8.34, p = .005, $\eta_{p}^{2}$ = .10). Again, the increase was stronger in the NT (F(1, 39) = 108.51, p <.001, $\eta_{p}^{2}$ = .74) than in the HT group (F(1, 39) = 46.65, p < .001, $\eta_{p}^{2}$ = .55) (Table S1).

During baseline the total number of “down” SBP ramps per minute was higher than the total number of “up” SBP ramps per minute in the entire sample (9.91 ± 3.46 vs. 8.03 ± 2.66, t(79) = -7.06, p < .001). In the NT group the number of “up” SBP ramps was higher than the number of “down” ramps during the task (11.58 ± 2.58 vs. 10.69 ± 2.53, t(39) = 2.22, p = .032); in contrast, in the HT group the number of “down” ramps was higher than the number of “up” ramps during the task (9.23 ± 2.55 vs. 8.25 ± 1.94, t(39) = -2.76, p = .009) (Table S1).

Overall, the slope of the SBP ramps was greater for the “up” than “down” ramps during baseline (t(79) = 7.71, p < .001 for reflex ramps; t(79) = 6.80, p < .001 for non-reflex ramps) and during the task period (t(79) = 8.67, p < .001 for reflex ramps; t(79) = 10.48, p <. 001 for non-reflex ramps). During baseline and task periods and for the “up” and “down” ramps, the slope was greater for reflex than non-reflex ramps (all ts(79) ≥ 2.44, all ps < .02) (Table S1).

The slope of the non-reflex SBP “up” ramps was greater in the NT than in the HT group (F(1, 78) = 6.90, p = .010, $\eta_{p}^{2}$ = .08). The slope increased between baseline and task periods (F(1, 78) = 121.99, p <.001, $\eta_{p}^{2}$ = .61) irrespective of group (interaction: F(1, 78) = 3.37, p = .070, $\eta_{p}^{2}$ = .04). The slope of the non-reflex SBP “down” ramps was also greater in the NT than in the HT group (F(1, 78) = 8.12, p = .006, $\eta_{p}^{2}$ = .09). It increased during the task (F(1, 78) = 93.81, p < .001, $\eta_{p}^{2}$ = .55) with a difference between groups (interaction: F(1, 78) = 6.43, p = .013, $\eta_{p}^{2}$ = .08). The increase was stronger in the NT group (F(1, 39) = 89.10, p < .001, $\eta_{p}^{2}$ = .70) than in the HT group (F(1, 39) = 22.00, p <. 001, $\eta_{p}^{2}$ = .36) (Table S1).

**Table S1**. Means (± SD) of the slope and amplitude of the blood pressure upstroke, total number of SBP “up” and “down” ramps per minute and the slope of the reflex and non-reflex SBP ramps during baseline and the arithmetic task in the hypotensive (HT) and normotensive (NT) groups.

|  |  | Baseline | Task |
| --- | --- | --- | --- |
| Slope of systolic upstroke (mmHg/ms) | HT | 0.46 ± 0.11 | 0.48 ± 0.11 |
|  | NT | 0.59 ± 0.15 | 0.65 ± 0.16 |
| SBP peak amplitude (mmHg) | HT | 46.73 ± 7.98 | 48.12 ± 8.31 |
|  | NT | 54.91 ± 9.98 | 58.25 ± 10.04 |
| nº “up” ramps (ramps/min) | HT | 6.80 ± 2.01 | 8.25 ± 1.94 |
|  | NT | 9.27 ± 2.68 | 11.58 ± 2.58 |
| nº “down” ramps (ramps/min) | HT | 8.69 ± 2.67 | 9.23 ± 2.55 |
|  | NT | 11.12 ± 3.75 | 10.69 ± 2.53 |
| Slope “up” reflex ramps (mmHg/s) | HT | 3.88 ± 1.05 | 5.86 ± 2.13 |
|  | NT | 5.86 ± 2.13 | 7.34 ± 2.30 |
| Slope “down” reflex ramps (mmHg/s) | HT | 3.22 ± 0.78 | 4.29 ± 1.13 |
|  | NT | 3.83 ± 0.86 | 5.57 ± 1.27 |
| Slope “up” non-reflex ramps (mmHg/s) | HT | 3.70 ± 1.31 | 5.08 ± 1.51 |
|  | NT | 4.07 ± 0.93 | 6.00 ± 1.33 |
| Slope “down” non-reflex ramps (mmHg/s) | HT | 3.13 ± 0.86 | 3.83 ± 0.94 |
|  | NT | 3.39 ± 0.81 | 4.59 ± 1.03 |

**S2. Number of reflex sequences per minute**

**S2.1. Cardiac branch**

The HT group exhibited a lower number of “up” (F(1, 78) = 15.06, p < .001, $\eta_{p}^{2}$ = .16) and “down” (F(1, 78) = 6.90, p = .01, $\eta_{p}^{2}$ = .08) sequences per minute than the NT group in the cardiac branch. The number of “down” sequences per minute decreased between baseline and task periods (F(1, 78) = 6.67, p = .01, $\eta_{p}^{2}$ = .08) with a difference between groups (interaction: F(1, 78) = 5.03, p = .028, $\eta_{p}^{2}$ = .06). While the change was significant in the NT group (F(1, 39) = 11.11, p = .002, $\eta_{p}^{2}$ = .22), “down” sequences remained unchanged in the HT group (F(1, 39) = 0.06, p = .81, $\eta_{p}^{2}$ = .002). In the entire sample more “up” than “down” sequences arose during the task (t(79) = 3.78, p < .001). However, this difference was seen in the NT group (t(39) = 4.39, p < .001) but not in the HT group (t(39) = 1.27, p = .21) (Table S2).

**S2.2. Vasomotor branch**

A lower number of “up” sequences per minute arose in the HT than in the NT group (F(1, 78) = 7.54, p = .007, $\eta_{p}^{2}$ = .09) in the vasomotor branch. A lower number of “down” sequences per minute was also detected in the HT than in the NT group, but this was only observed during baseline (t(78)=2.45, p =.017).The number of “down” sequences per minute increased between baseline and task as a function of group (interaction: F(1, 78) = 5.43, p = .02, $\eta_{p}^{2}$ = .07). The frequency of “down” sequences increased in the HT group (F(1, 39) = 10.02, p = .003, $\eta_{p}^{2}$ = .20) and remained unchanged in the NT group (F(1, 39) = 0.26, p = .62). Overall, there were more “down” than “up” sequences (t(79) = 3.58, p = .001 for baseline; t(79) = 6.59 p < .001, for task).

**S2.3. Myocardial branch**

The number of “up” (F(1, 78) = 25.42, p < .001, $\eta_{p}^{2}$ = .25) and “down” (F(1, 78) = 22.64, p < .001, $\eta_{p}^{2}$ = .23) sequences per minute in the myocardial branch was lower in the HT than in the NT group. The frequency decreased between baseline and task periods (F(1, 78) = 12.55, p = .001, $\eta_{p}^{2}$ = .14 for “up” sequences; F(1, 78) = 15.61, p <.001, $\eta_{p}^{2}$ = .17 for “down” sequences) with a difference between groups (interaction: F(1, 78) = 4.09, p = .047, $\eta_{p}^{2}$ = .05 for “up” sequences; F(1, 78) = 4.04, p = .05, $\eta_{p}^{2}$ = .05 for “down” sequences). The decrease was significant in the NT group (F(1, 39) = 10.28, p = .003, $\eta_{p}^{2}$ = .21 for “up” sequences; F(1, 39) = 12.08, p = .001, $\eta_{p}^{2}$ = .24 for “down” sequences) but not in the HT group (F(1, 39) = 2.34, p = .13, $\eta_{p}^{2}$ = .06 the “up” sequences; F(1, 39) = 3.56, p = .07, $\eta_{p}^{2}$ = .08 for “down” sequences). In the entire group there were more “up” than “down” sequences during the arithmetic task (t(79) = 2.17 p = .033) but not during baseline (t(79) = 1.84, p = .069) (Table S2).

**Table S2.** Means (± SD) of the number of “up” and “down” sequences per minute for the three baroreflex branches in the hypotensive (HT) and normotensive (NT) groups during baseline and the arithmetic task.

|  |  | Baseline | Task |
| --- | --- | --- | --- |
| Cardiac branch “up” sequences (sequences/min) | HT | 4.71 ± 2.26 | 5.15 ± 1.59 |
|  | NT | 6.37 ± 2.14 | 6.25 ± 1.54 |
| Cardiac branch “down” sequences (sequences/min) | HT | 4.91 ± 2.04 | 4.83 ± 1.73 |
|  | NT | 6.41 ± 2.43 | 5.27 ± 1.50 |
| Vasomotor branch “up” sequences (sequences/min) | HT | 2.09 ± 1.10 | 2.08 ± 0.87 |
|  | NT | 2.75 ± 1.22 | 2.48 ± 1.08 |
| Vasomotor branch “down” sequences (sequences/min) | HT | 2.61 ± 1.65 | 3.43 ± 1.50 |
|  | NT | 3.65 ± 2.12 | 3.48 ± 1.58 |
| Myocardial branch “up” sequences (sequences/min) | HT | 1.25 ± 2.03 | 0.99 ± 0.61 |
|  | NT | 2.79 ± 2.03 | 1.86 ± 1.16 |
| Myocardial branch “down” sequences (sequences/min) | HT | 1.14 ± 1.04 | 0.81 ± 0.83 |
|  | NT | 2.62 ± 2.01 | 1.62 ± 1.05 |

**S3. Baroreflex Effectiveness Index (BEI)**

**S3.1. Cardiac branch (cBEI)**

No group difference arose in cBEI (p = .30). cBEI decreased between baseline and task periods (F(1, 78) = 16.14, p < .001, $\eta_{p}^{2}$ = .17) as a function of group (interaction: F(1, 78) = 4.57, p = .03, $\eta_{p}^{2}$ = .06). The decrease in cBEI was significant in the NT (F(1, 39) = 36.84, p < .001, $\eta_{p}^{2}$ = .49) but not in the HT group (F(1, 39) = 1.16, p = .29, $\eta_{p}^{2}$ = .03) (Table S3).

**S3.2. Vasomotor branch (vBEI)**

No group difference was seen in vBEI (p = .77). vBEI changed between baseline and task periods as a function of group (interaction: F(1, 78) = 5.16, p = .03, $\eta_{p}^{2}$ = .06). vBEI decreased during task in the NT group (F(1, 39) = 4.98, p = .03, $\eta_{p}^{2}$ = .11) and remained unchanged in the HT group F(1, 39) = 1.45, p = .23, $\eta_{p}^{2}$ = .04) (Table S3).

**S3.3. Myocardial branch (mBEI)**

mBEI was lower in the HT group than in the NT group (F(1, 78) = 15.01, p < .001, $\eta_{p}^{2}$ = .16). Moreover, mBEI changed between baseline and task periods as a function of group (F(1, 78) = 5.25, p =.03, $\eta_{p}^{2}$ = .06). mBEI increased during the task in the HT group (F(1, 39) = 9.33, p = .004,$\eta_{p}^{2}$ = .19) and did not change in the NT group (F(1, 78) = 0.06, p = .81, $\eta_{p}^{2}$ = .002) (Table S3).

**Table S3**. Means (± SD) of the Baroreflex Effectiveness Index (BEI) during baseline and the arithmetic task in the hypotensive (HT) and normotensive (NT) groups for the cardiac (c), vasomotor (v) and myocardial branches of the baroreflex.

|  |  | Baseline | Task |
| --- | --- | --- | --- |
| cBEI (%) | HT | 60.11 ± 14.88 | 57.09 ± 11.25 |
|  | NT | 62.05 ± 7.42 | 52.02 ± 8.15 |
| vBEI (%) | HT | 33.37 ± 10.56 | 35.51 ± 8.10 |
|  | NT | 36.31 ± 8.86 | 33.48 ± 6.92 |
| mBEI (%) | HT | 20.85 ± 14.97 | 28.96 ± 12.13 |
|  | NT | 34.78 ± 17.18 | 34.10 ± 10.57 |

**S4. Associations between the magnitude of stimulation input and baroreflex function**

The overall number of SBP ramps per minute was inversely associated with the Baroreceptor reflex Sensitivity (BRS). This relationship was seen in both groups for the cardiac and vasomotor branches. In the myocardial branch the association was overall weaker, especially for the “down” ramps (as values of vBRS and mBRS are negative, positive correlations indicate inverse associations) (Table S4).

**Table S4.** Pearson correlations between the overall number of systolic blood pressure (SBP) “up” and “down” ramps and Baroreceptor reflex Sensitivity (BRS) in the cardiac (c), vasomotor (v) and myocardial (m) baroreflex branches in the hypotensive (HT) and normotensive (NT) groups during baseline (BL) and the arithmetic task (AT); * for p < .05, ** for p < .01.

|  |  | cBRS BL | cBRS AT | vBRS BL | vBRS AT | mBRS BL | mBRS AT |
| --- | --- | --- | --- | --- | --- | --- | --- |
| nº “up” ramps | HT | -.57** | -.52** | .30 | .40** | .38* | .37* |
|  | NT | -.34* | -.48** | .57** | .49** | .21 | .35* |
| nº “down” ramps | HT | -.75** | -.50** | .42** | .24 | .30 | .17 |
|  | NT | -.51** | -.51** | .56** | .37* | .29 | .30 |

The slope of reflex and non-reflex “up” and “down” SBP ramps was inversely associated with BRS in both groups. This association was closest for cBRS and smallest for mBRS (Table S5).

The slope of SBP ramps correlated positively with BEI. These associations were overall closer during baseline than during the arithmetic task period (Table S6).

**Table S5.** Pearson correlations between the slope of the systolic blood pressure (SBP) ramps and the Baroreceptor reflex Sensitivity (BRS) in the cardiac (c), vasomotor (v) and myocardial (m) baroreflex branches in the hypotensive (HT) and normotensive (NT) groups during baseline (BL) and the arithmetic task (AT); * for p < .05, ** for p < .01.

|  |  | cBRS BL | cBRS AT | vBRS BL | vBRS AT | mBRS BL | mBRS AT |
| --- | --- | --- | --- | --- | --- | --- | --- |
| Slope of “up”  reflex ramps | HT | -.49** | -.37* | .42** | .12 | .16 | .24 |
|  | NT | -.59* | -.39* | .42** | .18 | .34* | .21 |
| Slope of “down”  reflex ramps | HT | -.59** | -.43** | .32* | .16 | .29 | .19 |
|  | NT | -.49** | -.40* | .30 | .40* | .30 | .15 |
| Slope of “up”  non-reflex ramps | HT | -.42** | -.44** | .21 | .26 | .37* | .13 |
|  | NT | -.55** | -.55** | .35* | .28 | .44** | .21 |
| Slope of “down”  non-reflex ramps | HT | -.59** | -.47** | .39* | .17 | .31 | .24 |
|  | NT | -.48** | -.60** | .28 | .37* | .42** | .30 |

**Table S6.** Pearson correlations between the slope of systolic blood pressure (SBP) ramps and the Baroreflex Effectiveness Index (BEI) in the cardiac (c), vasomotor (v) and myocardial (m) baroreflex branches in the hypotensive (HT) and normotensive (NT) groups during baseline (BL) and the mental arithmetic task (AT); * for p < .05, ** for p < .01.

|  |  | cBEI BL | cBEI AT | vBEI BL | vBEI AT | mBEI BL | mBEI AT |
| --- | --- | --- | --- | --- | --- | --- | --- |
| Slope of “up”  reflex ramps | HT | .47** | .38* | .40* | .-06 | -.11 | -.07 |
|  | NT | .43** | .30 | .40* | .15 | .45** | .31* |
| Slope of “down”  reflex ramps | HT | .23 | .25 | .36* | -.22 | .02 | .03 |
|  | NT | .43** | -.27 | .34* | .-01 | .42** | .24 |
| Slope of “up” non  reflex ramps | HT | .48** | .09 | .31 | .-23 | .10 | -.17 |
|  | NT | .55** | -.01 | .30 | .03 | .42** | .35* |
| Slope of “down”  non reflex ramps | HT | .42** | .20 | .37* | -.22 | .01 | .08 |
|  | NT | .40* | .10 | .25 | .13 | .48** | .27 |

The slope of BP rise during the systolic peak correlated positively with cBRS in the HT group during baseline and task periods. The negative correlation with mBRS in both groups during baseline implies a positive association between sensitivity and the slope of the SBP peak. The positive correlation with vBRS in both groups implies an inverse association between baroreflex sensitivity and the slope to the systolic peak (values of vBRS and mBRS are negative such that positive correlations indicate inverse associations) (Table S7).

**Table S7.** Pearson correlations between the slope of the BP rise suring the systolic peak and Baroreceptor reflex Sensitivity (BRS) for the cardiac (c), vasomotor (v) and myocardial (m) baroreflex branches in the hypotensive (HT) and normotensive (NT) groups during the baseline (BL) and the arithmetic task (AT) ; * for p < .05, ** for p < .01.

|  |  | cBRS BL | cBRS AT | vBRS BL | vBRS AT | mBRS BL | mBRS AT |
| --- | --- | --- | --- | --- | --- | --- | --- |
| Slope SBP peak | HT | .43** | .33* | .47** | .47** | -.40* | -.08 |
|  | NT | -.25 | -.39* | .42** | .37* | -.39* | -.12 |

**S5. Differences in baroreflex sensitivity and effectiveness between “up” and “down” sequences**

**S5.1. Cardiac branch**

The Group x Condition interaction for cBEI was significant regarding “up” sequences (F(1, 78) = 4.67, p = .03, $\eta_{p}^{2}$ = .06) but not “down” sequences (F(1, 78) = 2.90, p = .09, $\eta_{p}^{2}$ = .04). cBEI for “up” sequences decreased during the task in the NT group (F(1, 39) = 29.12, p < .001, $\eta_{p}^{2}$ = .43) but not in the HT group (F(1, 39) = 0.77, p = .39, $\eta_{p}^{2}$ = .02). Regardless of group, cBEI was higher for “up” than “down” sequences during baseline (67.53 ± 16.08 vs. 56.22 ± 11.32; t(79) = 6.98, p < .001) and task (58.94 ± 14.45 vs. 50.79 ± 10.09; t(79) = 5.24, p < .001) periods. cBRS was higher in the “down” than “up” sequences during the baseline (18.71 ± 9.19 vs. 17.66 ± 8.40; t(79) = -2.08, p = .041) but not during the task (12.03 ± 5.34 vs. 11.73 ± 5.25; t(79) = -.63 p = .53) (Table S8).

**S5.2. Vasomotor branch**

While for “up” sequences vBRS remained unchanged during the task (-12.41 ± 7.01 vs. -10.94 ± 6.67; F(1, 78) = 2.36, p = .13, $\eta_{p}^{2}$ = .03, for baseline and task, respectively), it decreased for “down” sequences (-13.57 ± 10.38 vs. -10.49 ± 5.04; F(1, 78) = 7.35, p =.008, $\eta_{p}^{2}$ = .09). The Group x Condition interaction for vBEI (see S3.2) was mainly driven by the “down” sequences (Condition effect: (F(1, 78) = 5.71, p = .018, $\eta_{p}^{2}$ = .07; interaction: F(1, 78) = 4.92, p = .02, $\eta_{p}^{2}$ = .06). vBEI increased for “down” sequences during the task in the HT group (F(1, 39) = 8.94, p = .005, $\eta_{p}^{2}$ = .19) but not in the NT group (F(1, 39) = 0.02, p =.89). Regarding vBEI for “up” sequences the Condition effect was significant (F(1, 78) = 15.89, p < .001, $\eta_{p}^{2}$ = .17) but not the interaction Group x Periods (F(1, 78) = .09, p =.77, $\eta_{p}^{2}$ < .01). During the task vBEI was higher for “down” than “up” sequences (39.12 ± 11.61 vs. 28.82 ± 9.54; t(79) = -6.40, p < .001); this did not apply for the baseline (34.66 ± 14.26 vs. 34.97 ± 11.61 for “down” and “up” sequences, respectively; t(79) = 0.16, p = .98).

**S5.3. Myocardial branch**

The group difference in mBRS differed as a function of sequence type. While for “up” sequences the group difference was not significant (F(1, 78) = 3.27, p = .07, $\eta_{p}^{2}$ = .04), for “down” sequences the HT group exhibited greater mBRS than the NT group (F(1, 78) = 6.89, p = .01, $\eta_{p}^{2}$ = .08) (Table S8). The Group x Condition interaction for mBEI (see 3.3) was mainly driven by the “up” sequences; this interaction was significant for “up” (F(1, 78) = 8.19, p =.005, $\eta_{p}^{2}$ = .10) but not “down” (p = .64) sequences. For “up” sequences mBEI increased during the task in the HT group (F(1, 39) = 7.10, p = .01, $\eta_{p}^{2}$ = .15) but remained unchanged in the NT group (F(1, 39) = 1.96, p = .17, $\eta_{p}^{2}$ = .06). In the entire sample, mBRS was higher for “down” than “up” sequences (-2.64 ± 1.43 vs. -2.21 ± 1.11; t(79) = 2.15, p = .03 for baseline; -2.62 ± 1.76 vs. -2.05 ± 1.29; t(79) = 2.59, p = .012 for task). mBEI was higher for “up” than “down” sequences (36.99 ± 21.01 vs. 27.54 ± 15.76; t(79) = 4.41, p < .001 for baseline; 39.17± 15.03 vs. 30.53±12.81; t(79) = 3.62, p = .001 for task).

**Table S8.** Means (± SD) of Baroreflex Effectiveness Index (BEI) and Baroreceptor reflex Sensitivity (BRS) during baseline and the arithmetic task in the variables in which differences arose as a function of SBP increase (“up” sequences) and SBP decrease (“down” sequences); c = cardiac branch, v = vasomotor branch, m = myocardial branch.

|  |  | Baseline | Task |
| --- | --- | --- | --- |
| cBEI “up” (%) | HT | 66.42 ± 19.12 | 62.97 ± 15.92 |
|  | NT | 68.64 ± 12.47 | 54.89 ± 11.64 |
| cBEI “down” (%) | HT | 55.09 ± 13.95 | 52.09 ± 10.56 |
|  | NT | 57.36 ± 7.89 | 49.50 ± 9.56 |
| vBEI “up” | HT | 34.90 ± 13,06 | 29.22 ± 9.46 |
|  | NT | 35.04 ± 10.13 | 28.44 ± 9.72 |
| vBEI “down” | HT | 32.54 ± 14.54 | 41.15 ± 11.91 |
|  | NT | 36.78 ± 13.83 | 37.10 ± 11.09 |
| mBRS “up” | HT | -2.30 ± 1.28 | -2.30 ± 1.57 |
|  | NT | -2.12 ± 0.89 | -1.79 ± 0.88 |
| mBRS “down” | HT | -2.77 ± 1.63 | -3.13 ± 2.17 |
|  | NT | -2.51 ± 1.18 | -2.11 ± 1.00 |
| mBEI “up” | HT | 29.71 ± 18.77 | 39.10 ± 14.40 |
|  | NT | 44.29 ± 20.81 | 39.23 ± 14.82 |
| mBEI “down” | HT | 23.48 ± 13.98 | 27.49 ± 12.96 |
|  | NT | 31.60 ± 16.56 | 33.57 ± 12.06 |

**Comments**

During baseline “down” SBP ramps were more frequent than “up” SBP ramps in both study groups. However, during the task period the NT group exhibited more “up” than “down” SBP ramps, whereas the HT group exhibited more “down” than “up” SBP ramps. This difference is congruent with the greater increase in BP during the task in the NT group, which produce more “up” than “down” SBP changes.

The slope of the reflex and non-reflex SBP ramps was greater in the NT than in the HT group and increased during the arithmetic task. The slope increase was stronger in the NT than in the HT group. Moreover, slope was greater for “up” than “down” ramps and for reflex than non-reflex ramps (see Reyes del Paso et al., 2006 [1] for similar observations).

The number of SBP ramps and their slope were inversely associated with BRS. In contrast, especially during baseline, the slope of SBP ramps correlated positively with BEI (see Reyes del Paso et al., 2006 [1] for similar observations). These associations were not seen in the HT group for the myocardial branch, suggesting alterations in the baroreflex control of inotropic function (see main manuscript). More frequent SBP ramps and steeper slope of the ramps involve greater stimulation input to the baroreceptors, leading to a larger number of reflex sequences (i.e., higher BEI). Steeper slope of SBP ramps is accompanied by greater stretch of the vascular tissue and thus stronger baroreceptor activation and higher probability of reflex responses.

The inverse association between the slope of SBP ramps and BRS may be due to the quantification of BRS, i.e. its definition as change in the corresponding response variable (e.g., interbeat interval, IBI, for cBRS) per unit of SBP change. Though a steeper ramp leads to greater absolute change in IBI than a less steep one, when expressed in changes per unit of mmHg, the magnitude of BRS (ms/mmHg) can be lower. In terms of a compensatory mechanism, BRS may decrease during steeper ramps. For example, in a relatively steep ramp with a difference of 10 mmHg between the first and last beat, the absolute IBI change for a cBRS value of 17 ms/mmHg would be 170 ms. In a less steep ramp with a difference of 5 mmHg between the first and last beat, the absolute IBI change for a cBRS value of 20 ms/mmHg would be 100 ms.

The slope of BP rise to the systolic peak was positively associated with cBRS in the HT group. While the slope of the systolic peak also correlated positively with mBRS in both groups during baseline, it correlated negatively with vBRS. This pattern of correlations suggests different behaviors of the heart-related and vasomotor baroreflex branches. The associations for cBRS are in accordance with observations of Eckberg (1977) [2] in young healthy man, suggesting that variations in the rate of carotid pressure change influence the baroreflex responses. However, the influence seems to be minimal within the BP normal range, which may explain the restriction of the positive association to the HT group. For the myocardial branch, the positive association may be present in a larger BP range including hypotension and normotension. The inverse association between the slope of the systolic peak and vBRS might have an adaptive function. During decrease of vessel diameter BP and pulse wave velocity increase. Given the positive association between vBRS and total peripheral resistance (TRP), the reduction of vBRS in case of high slope of the systolic peak allows for peripheral vasodilatation, and thus decrease of BP and pulse velocity which compensate the greater BP slope rise. This may by a protective mechanism against vascular damage during steep SBP peaks.

A lower number of reflex sequences for all three baroreflex branches arose in the HT than in the NT group. The effect size of the difference was largest for the myocardial branch. It may be argued that hypotension is associated with lower BP variability (i.e., less BP ramps) and generation of fewer reflex sequences. In turn, lower BP variability may result from higher BRS. In a previously study in healthy subjects, more sequences were observed in the cardiac than in the vasomotor and myocardial branches [3]. In the HT group, the number of “down” sequences in the vasomotor branch increased during the arithmetic task, reflecting greater activity of the baroreflex in buffering BP decreases by increases in vasomotor tone. The overall higher number of “down” than “up” sequences and the higher number of “up” than “down” sequences specifically during the arithmetic task in the myocardial branch corroborate previous observations [3].

While no group difference arose for cBEI, this parameter decreased during the task in the NT group but not in the HT group. Apparently, the reflex maintained its higher capacity in compensating BP rises by increasing vagal activity during the task in hypotension. The lack of cBEI change in the HT group accords with the lower BP, heart rate (HR) stress-reactivity and the lack of task-induced HRV change in hypotension. In the NT group, but not in the HT group, cBEI for “up” sequences decreased during the task, indicating that the reflex maintained its effectiveness in compensating BP increases in hypotension. The overall cBEI decrease in normotensive individuals during the task allowed for greater BP and HR increases. In the entire sample cBEI was higher for the “up” than “down” sequences, reflecting greater effectiveness of the reflex in buffering BP increases (by vagal activation) than decreases (by vagal inhibition) [1, 3-4]. Accordingly, close positive associations of cBRS with IBI and HRV were seen in both groups.

vBEI did not differ between groups but changed during the task as a function of group. While vBEI for the “up” sequences decreased in both groups, vBEI for the “down” sequences increased in the HT group but not in the NT group. This suggest that the reflex was more effective in buffering BP decreases by reflex increases in vasomotor tone in hypotensive individuals, thereby facilitating task-induced BP increase by the actuation of the vasomotor brach. In the same vein, the number of “down” sequences increased during the task in the HT group. In contrast, vBEI for the “up” sequences decreased during the task in both groups.

The decrease in vBRS during the task only reached significance for the “down” sequences. This indicates that the reflex maintained its sensitivity in buffering BP increases by reductions in α-adrenergic vasoconstriction but reduced its sensitivity in buffering BP decreases, thus favoring lower BP increases during stress. While the reflex became less effective in buffering BP increases by TPR reduction in both groups (mBEI) (thus favoring the maintenance of greater TPT and BP), in the HT group the effectiveness in buffering BP decrease rose during the task, thus facilitating BP increase and suggesting an optimal functioning of the vasomotor branch in hypotension.

The higher number of “down” than “up” sequences as well as the higher vBEI in the “down” than in “up” sequences are consistent with previous studies and the notion of certain functional asymmetry of the baroreflex in its vasomotor branch, with greater protection of BP falls (by activating α-adrenergic influences to increase vascular tone) than BP rises (by reducing vascular tone) [3]. This lead to positive associations between vBRS and TPR and TPR variability.

Though mBRS was overall higher in the HT group than in the NT group, the difference varied as a function of sequence type; it reached significance for the “up” sequences but for the “down” sequences. mBEI was overall lower in the HT group than in the NT group; moreover, for the “up” sequences mBEI increased during the task in the HT group but remained unchanged in the NT group. This suggests that the reflex became more effective in producing compensatory stroke volume (SV) reductions following BP increases in hypotension. Notably, though in the HT group the reflex compensated BP fluctuations by greater SV changes (mBRS), it was overall less effective in eliciting SV responses to these fluctuations (mBEI).

In the entire sample, mBRS was higher in the “down” than in the “up” sequences. This contrasts with a previous observation of no difference between mBRS for “up” and “down” sequences both computed from pre-ejection period and SV [3]. However, the observation of greater mBEI in “up” than “down” sequences is in line with this study.

**References**

1. Reyes del Paso GA, Hernández JA, González MI (2006) Differential evaluation of the baroreceptor cardiac reflex effectiveness as a function of sequence length. Int J Psychophysiol 59:91–96

2. Eckberg DL (1977) Baroreflex inhibition of the human sinus node: importance of stimulus intensity, duration, and rate of pressure change. J Physiol 269:561–577

3. Reyes del Paso GA, de la Coba P, Martín-Vázquez M, Thayer JF (2017) Time domain measurement of the vascular and myocardial branches of the baroreflex: A study in physically active versus sedentary individuals. Psychophysiology 54:1528–1540

4. Reyes del Paso GA, González I, Hernández JA (2004) Baroreceptor sensitivity and effectiveness varies differentially as a function of cognitive-attentional demands. Biol Psychol 67:385–395
